# Epidermolysis bullosa: Advances in research and treatment

**DOI:** 10.1111/exd.13979

**Published:** 2019-08-08

**Authors:** Christine Prodinger, Julia Reichelt, Johann W. Bauer, Martin Laimer

**Affiliations:** ^1^ EB House Austria Research Program for Molecular Therapy of Genodermatoses Department of Dermatology University Hospital of the Paracelsus Medical University Salzburg Salzburg Austria; ^2^ Department of Dermatology University Hospital of the Paracelsus Medical University Salzburg Austria; ^3^ Department of Dermatology Venereology and Allergology, Medical University of Innsbruck Innsbruck Austria

**Keywords:** calcipotriol, diacerein, gene therapy, genodermatoses, squamous cell carcinoma

## Abstract

Epidermolysis bullosa (EB) is the umbrella term for a group of rare inherited skin fragility disorders caused by mutations in at least 20 different genes. There is no cure for any of the subtypes of EB resulting from different mutations, and current therapy only focuses on the management of wounds and pain. Novel effective therapeutic approaches are therefore urgently required. Strategies include gene‐, protein‐ and cell‐based therapies. This review discusses molecular procedures currently under investigation at the EB House Austria, a designated Centre of Expertise implemented in the European Reference Network for Rare and Undiagnosed Skin Diseases. Current clinical research activities at the EB House Austria include newly developed candidate substances that have emerged out of our translational research initiatives as well as already commercially available medications that are applied in off‐licensed indications. Squamous cell carcinoma is the major cause of death in severe forms of EB. We are evaluating immunotherapy using an anti‐PD1 monoclonal antibody as a palliative treatment option for locally advanced or metastatic squamous cell carcinoma of the skin unresponsive to previous systemic therapy. In addition, we are evaluating topical calcipotriol and topical diacerein as potential agents to improve the healing of skin wounds in EBS patients. Finally, the review will highlight the recent advancements of gene therapy development for EB.

## 1 EPIDERMOLYSIS BULLOSA

Epidermolysis bullosa (EB) encompasses a group of rare, clinically and genetically heterogeneous genodermatoses with an estimated 500 000 cases worldwide.^1^ It is characterized by moderate to excessive fragility of epithelial tissues with prototypic blistering or erosions following minimal trauma (mechanobullous dermatoses).

Epidermolysis bullosa is caused by mutations involving at least 20 genes coding for components of the cytoskeletal keratin intermediate filaments, cell junctions such as, desmosomes and hemidesmosomes, and other molecules contributing to intraepidermal adhesion and dermo‐epidermal anchorage of skin and mucous membranes.^2^, ^3^, ^4^, ^5^ Recently, mutations in the *KLHL24* (Kelch‐like family member 24) gene encoding for a component of a ubiquitin‐ligase complex have been delineated to underlie subvariants of autosomal dominant EB simplex.^6^, ^7^, ^8^ The mutant protein is more stable owing to the absence of auto‐ubiquitination and promotes excessive ubiquitination and degradation of keratin 14 (K14). In addition, a homozygous splicing mutation in CD151, encoding a tetraspanin expressed in the basement membrane zone, was very recently found in a patient with a Kindler syndrome‐like phenotype (early blistering subsiding with age, multi‐systemic involvement including nephropathy).^9^ The same group also revealed homozygous missense mutations in PLOD3 encoding lysyl hydroxylase 3 (LH3) to cause widespread connective tissue abnormalities including extensive joint contractures, skeletal abnormalities, reduced growth and sublamina densa skin blistering similar to that in recessive dystrophic EB.^10^ Pathogenetically, a deficient glycosylation activity is implicated to alter post‐translational modifications of type VII and other collagens causing deleterious changes in deposition and organization of extracellular matrix, such as a deficient extracellular assembly of anchoring fibrils that compromises their stability and renders them subject to degradation.

These molecular aberrations finally impair the structural and functional integrity within the highly specialized interfaces, which are crucial for cell adhesion, proliferation and differentiation, tissue repair, and barrier function.^2^, ^3^, ^5^ Consequently, this leads to characteristic diminished resistance to mechanical stress and shearing forces with subsequent cell and tissue damage.

The index genes involved are also partly expressed in other epithelialized (gastrointestinal, respiratory, urogenital tract) or mesenchymal (skeletal muscle) organs. Apart from secondary extracutaneous involvement, this also explains the occurrence of primary extracutaneous manifestations and relevant complications especially in the severe forms of EB, making them a multisystem disease with significant morbidity and mortality.^11^

Type (homo‐ vs heterozygosity), number (monogenic, digenic inheritance) and location of mutation(s) within the gene or gene segment, as well as the spectrum of subsequent quantitative (absence, reduction) or qualitative (gradual loss of function) disruption of protein expression, result in considerable genetic heterogeneity with complex genotype‐phenotype correlations.^12^, ^13^ In addition to the primary structural‐functional genetic defect, secondary epigenetic and biochemical (eg differentially regulated expression of a host of other genes involved in the maintenance and function of this microenvironment, induction of inflammatory cascades) or environmental factors also have a major impact on the individual phenotype.^12^, ^13^ This corresponds to a considerably broad spectrum of clinical manifestations and severity, ranging from limited moderate blistering on primarily mechanically exposed sites such as hands and feet that manifests at times only in late childhood or adolescence, to extensive, generalized, fatal (multiorgan) involvement with (seemingly spontaneous) blistering affecting most of epithelialized tissues already at birth. Common causes of (early) death include malnutrition, infections, organ failure and skin cancer.^14^

Currently, no general curative therapy is available for all EB types. In light of the morbidity as well as lethality of numerous subvariants as well as the current availability of largely only symptomatic treatment options, causative therapeutic approaches are therefore urgently required. Such strategies include gene‐, protein‐ and cell‐based therapies.^15^ This review discusses molecular procedures currently under investigation at the EB House Austria (http://www.eb-haus.org), a designated Centre of Expertise implemented in the European Reference Network for Rare and Undiagnosed Skin Diseases (ERN Skin) with currently 56 partners (http://skin.ern-net.eu/ ), and at other centres. Albeit still largely experimental and facing major hurdles including safety and efficiency to overcome, these techniques should contribute to determine curative perspectives.

## 2 CLINICAL RESEARCH

As stated above, the severe forms of EB are not restricted to the skin but affect the entire body. Several approaches have therefore been developed in the quest for a systemic therapy. Attempts using allogeneic bone marrow transplantation (BMT) with or without infusion of mesenchymal stem cells (MSCs) have shown some transient beneficial clinical response in a small number of patients with RDEB^16^, ^17^, ^18^, ^19^ but not in patients with JEB.^20^ The mortality rates that come with such serious interventions are considerable, and the initially high expectations for the approach have not been met.

Several substances that elicit premature termination codon (PTC) read‐through showed promising results in vitro for specific RDEB‐ and JEB‐associated mutations.^21^, ^22^, ^23^ The promise of PTC read‐through substances, which often fall into the small molecule category of drugs available for repurposing, is to express a previously absent protein. The antibiotic gentamicin has already been tested in a few patients with PTC mutations and seemed to improve the skin phenotype for a few months when applied topically or via intradermal injections in some patients.^24^ Clinical studies to test the effect of intravenous injections of gentamicin are currently conducted for RDEB (NCT03392909) and JEB (NCT03526159).

Systemic recombinant human type VII collagen protein administration is another therapeutic approach that is pursued for the treatment of RDEB patients. It has previously shown promising results in a mouse model for RDEB^25^ and is currently being tested in a phase I/II randomized controlled single‐blind clinical trial (NCT03752905) at Stanford University.

Current clinical research activities at the EB House Austria include the development of novel therapies that have emerged out of our translational research initiatives, as well as commercially available medications that are used for “off‐label” uses.

### 2.1 Nivolumab in locally advanced/metastatic squamous cell carcinoma

In a prospective, multi‐centre, phase II trial (Eudra CT‐No. 2016‐002811‐16), we are evaluating the administration of nivolumab (an anti‐PD1 monoclonal antibody) for the palliative treatment of patients with DEB who have locally advanced or metastatic squamous cell carcinomas of the skin (SCCS) that have been unresponsive to other systemic therapies. The target population also includes patients suffering from generalized severe recessive dystrophic epidermolysis bullosa, in which SCCS, owed to a highly aggressive course with early metastatic spread, is the primary cause of death with a cumulative risk of 70% to die by age 45.^26^

For both EB and non‐EB SCCS, evidence on overall clinical effectiveness of standard chemotherapeutic agents (with rather poor tolerability among older patients) as well as systemic therapies targeting the epidermal growth factor receptor (EGFR) pathway remains mostly limited and is often of short duration.^26^, ^27^, ^28^

Immunosuppression plays a major role in the pathogenesis of SCCS,^29^, ^30^, ^31^, ^32^ which is evident especially in solid organ transplant patients as well as severe EB forms along with malnutrition, anaemia and chronic infections.^33^, ^34^ Tumor emergence and progression may further depend upon acquisition of traits that allow cancer cells to evade immune surveillance and an effective immune response.^35^, ^36^, ^37^

Against this background, current immunotherapy efforts attempt to break the apparent tolerance of the immune system to tumor cells and antigens by either introducing cancer antigens by therapeutic vaccination or by modulating regulatory checkpoints of the immune system.^38^, ^39^, ^40^ As for the latter, blockade of programmed death receptor‐1 (PD‐1) was shown to impair downregulation of T‐cell activation upon PD‐1 binding^28^, ^29^ and proved clinical activity in a variety of tumor types, including melanoma, renal cell cancer and non‐small cell lung cancer.^41^

Plausible molecular features of SCCS,^42^, ^43^, ^44^ consistent data from animal studies^45^ as well as beneficial evidence from clinical trials in advanced head and neck SCC (HNSCC),^46^, ^47^ and, recently, locally advanced/metastatic SCCS^48^, ^49^, ^50^ support the therapeutic scope of PD‐1 inhibition. Objective response was reported to be approximately 50% in early phase I and II trials,^49^ leading to the approval of the PD‐1 inhibitor cemiplimab for the treatment of patients with metastatic or locally advanced SCCS who are not candidates for curative surgery or curative radiation.

Despite obvious progress, current knowledge to correlate clinical effectiveness of checkpoint inhibitors with the tumor immune microenvironment is still limited in SCCS and should be subject to further investigation.^48^, ^51^, ^52^, ^53^, ^54^, ^55^

Our trial seeks to determine the objective response rate of immunotherapy with nivolumab in index patients with using Response Criteria in Solid Tumors version 1.1 (RECIST1.1) per site assessment up to 2 years as the primary objective. Treatment efficacy will be correlated with the number of tumor‐infiltrating lymphocytes (TILs) and CD8‐positive lymphocytes at baseline.

### 2.2 Topical calcipotriol in dystrophic epidermolysis bullosa

Enhanced tissue fragility, persistent wounds and inflammation in EB predispose to complications including infections, sepsis and the aforementioned development of aggressive SCCS.^1^, ^56^

Common wound colonizers in EB, such as *Staphylococcus sp., Streptococcus sp., Candida sp*. and *Pseudomonas aeruginosa,* 57 with loss of microbial diversity, may contribute to prolonged inflammation, delayed wound healing and chronic wounding.^58^

Most recently, a role for innate immune sensing of microbial products, such as bacterial flagellin via toll‐like receptor 5, in promoting wounding‐ and inflammation‐induced skin tumorigenesis was demonstrated,^59^ highlighting that local antisepsis, topical antibiotics and local wound care are critically important in wound management and possibly in cancer prevention in EB.

All currently existing approaches for the treatment of bacterial infected wounds have some disadvantages. Antiseptic baths, which are recommended for dystrophic EB patients, are often time‐consuming and exhausting as well as painful for the patients since all dressings have to be carefully removed. Despite beneficial results, silver‐containing creams (eg Flammazine) should not be applied for more than maximal 2‐4 weeks due to emerging silver toxicities attributable to systemic absorption. Finally, long‐lasting application of antibiotic ointments for the treatment of infected wounds may lead to multi‐resistant bacterial strains, such as methicillin‐resistant *Staphylococcus aureus*.

Antimicrobial peptides (AMPs) form part of the body's innate immune response, serve as potent antibacterial substances that control pathogenic infections and activate the innate and adaptive immune system.^60^ Cathelicidin (human cationic antimicrobial protein 18, hCAP18) is a prominent AMP in human epithelial cells, which serves to augment host defense, and appears to play a role in tissue repair and wound closure.^61^, ^62^, ^63^, ^64^ In response to cutaneous infections, hCAP18 is upregulated in skin and shows direct antimicrobial, antiviral and antifungal activity.^64^

Cathelicidin is directly regulated by vitamin D.^65^ Sun exposure, leading to production of the prehormone in the skin, serves as a distinct source of vitamin D. In EB patients, limited sun exposure due to wound dressings and reduced outdoor activity may lead to vitamin D deficiency,^66^ resulting in reduced cathelicidin production and lowered antimicrobial defenses.

Our preliminary data on hCAP18 mRNA levels in skin tissue samples taken from RDEB patients and RDEB patient‐derived keratinocyte cell lines support this hypothesis.^67^ We also observed an increase in hCAP18 expression at transcript and protein level in RDEB keratinocytes by treatment with distinct concentrations of the active vitamin D3 analogue, calcipotriol, which is already used routinely in the clinical treatment of psoriasis.^67^, ^68^ This correlated with reduced colony formation upon incubation of *E coli* with supernatants from cultured calcipotriol‐treated RDEB cells compared to controls while retaining any anti‐proliferative effect of calcipotriol detrimental to wound healing.^67^, ^69^

Based on our results including a promising single‐patient observation study,^67^ we are currently investigating the potential of improved wound healing in dystrophic EB via increased in vivo expression of cathelicidin and thus enhanced antimicrobial defense by topical administration of calcipotriol in a double‐blind, placebo‐controlled crossover study (EudraCT‐No 2016‐001967‐35). Notably, locally produced cathelicidin can serve as a chemoattractant for innate immune cells including neutrophils, which produce and store vast amounts of AMPs and release them at sites of infection,^70^ underlining the utility of this approach in diseases characterized by increased susceptibility to bacterial infections. The immunomodulatory effects should foster the restoration of microbial diversity and inflammatory balance in EB skin. Moreover, increased production of LL‐37 (the active peptide form of cathelicidin) upon vitamin D treatment has previously been reported in chronic and non‐healing wounds.^62^

### 2.3 Topical diacerein in EB simplex

We and others have recently shown a significant constitutive upregulation of interleukin‐1beta (IL‐1ß) in keratinocytes of patients with severe generalized EB simplex (EBS‐gen sev), an autosomal dominant subvariant based on mutations within the keratin genes *KRT5* or *KRT14*, with subsequent disintegration and collapse of the intermediate filament network, characteristic protein aggregation and increased fragility to mechanical stress and osmotic shock.^71^, ^72^, ^73^ Increased IL‐1ß activity results in the activation of the c‐Jun N‐terminal‐kinase (JNK)/mitogen‐activated protein kinase (MAPK) stress pathway and subsequently, via induction of downstream transcription factors, in the overexpression of K14, JNK and IL‐1β itself in a positive feedback loop.^73^

This mechanism is suspected to increase the expression of the dominantly interfering mutated K14 allele, thereby potentially aggravating the EBS‐gen sev phenotype as compared to milder subtypes of EBS caused by nonsense mutations within the same gene. Additionally, blister formation was found to be related to matrix metalloproteinase (MMP)‐9 and chemokine CXCL8/IL8, whose expression is also dependent on the IL‐1β signalling pathway, that thereby modulates the tissue microenvironment.^74^, ^75^ In this context, previous studies revealed high levels of IL‐1β in blister fluid and serum samples from EBS patients^76^, ^77^ and significantly upregulated IL‐1β in a mouse model for EBS.^78^ In summary, studies on EBS‐gen sev keratinocytes’ transcriptomes and proteomes revealed a complex landscape of dysregulation, triggered by a single, heterogeneous point mutation within either of the basal keratins, and prominently affecting downstream transcriptional targets of the IL‐1ß‐induced stress pathway.

Therapeutic approaches to inhibit IL‐1β could thus be beneficial to patients through reduction of downstream cellular inflammation. Indeed, treatment of EBS‐gen sev patient keratinocytes with diacerein, a small molecule component of rhubarb root and reported IL‐1β inhibitor, not only reduced the expression levels of K14 and IL‐1β and the phosphorylation levels of JNK, but also stabilized the intermediate filament network upon heat shock.^73^ Additionally, the invasive potential of EBS‐gen sev keratinocytes decreased upon IL‐1ß inhibition in functional assays, presumably because of reduced matrix metalloproteinase expression and increased expression of cell‐junction proteins. Our data are in line with the former demonstration of reduced overall K14 expression levels upon mRNA correction via spliceosome‐mediated RNA *trans*‐splicing (SMaRT), which shifts the expression ratio of wild‐type vs mutated K14 alleles towards wild type.^79^

Based on our in vitro data, we launched a double‐blind, randomized, placebo‐controlled phase 1 pilot study of topical diacerein 1% cream in 5 patients with EBS‐gen sev.^80^ Topical formulation of oral diacerein (which is already approved for the treatment of osteoarthritis) should account for feasibility and future cost‐efficiency while minimizing the probability of systemic side effects. The trial design consisted of a 6 weeks open phase, which was followed by a randomized treatment withdrawal, where one armpit of each patient was assigned to placebo and one to diacerein in a double‐blind manner. Consequently, all patients could benefit from the treatment during the open phase and in‐patient placebo controls would compensate for individual differences in blister numbers. The number of blisters was reduced significantly (mean: −78% of baseline) within 2 weeks and remained significantly below the initial level even during withdrawal (suggesting a carry‐over effect of the foregoing treatment). No adverse effect was reported. Confirmative (monocentric and international multi‐centre) phase II/III studies to assess the potential of topical diacerein to downregulate the activity of IL‐1 and reduce the auto‐inflammatory effects in the skin of patients with EBS have been recently published.^81^ In this 2‐period crossover trial, 17 patients were randomized to either placebo or diacerein for a 4‐week treatment and a 3‐month follow‐up in period 1. After a washout, patients were crossed over during period 2. Of the patients receiving diacerein, 86% in episode 1% and 37.5% in episode 2 met the primary end point (vs 14% and 17% with placebo, respectively), which was prespecified as the proportion of patients with a reduction of number of blisters by more than 40% from baseline in selected areas over the treatment episode. This effect was still significant after the follow‐up. Notably, changes in absolute blister numbers were significant for the diacerein group only. No adverse effects were observed.

While the low patient numbers and no invasive data acquisition because of clinical burden in children pose relevant limitations, this trial provides evidence of the impact of 1% diacerein cream in the treatment of EBS‐gen sev.

### 2.4 Gene therapy

So far, only a small number of EB patients have undergone gene therapy. These individuals received the classical treatment approach termed replacement gene therapy. The treatment, which is suitable for recessive forms of EB, relies on ex vivo viral transfer of a functional cDNA copy of the defective endogenous gene into keratinocytes and/or fibroblasts isolated from skin biopsies, which are then grafted back onto the skin. The approach has first been applied in a patient with junctional EB caused by mutations in *LAMB3* 82 and has since been used in two additional JEB patients.^83^, ^84^ Most recently treated patients have undergone good manufacturing practice (GMP)‐guided ex vivo gene therapy in collaboration with the Center for Regenerative Medicine in Modena, Italy (Tables 1 and 2). Two phase I/II trials, one of which for recessive dystrophic EB (*COL7A1*, EudraCT‐No 2015‐004592‐74) and the other one for junctional EB (*COL17A*, EudraCT‐No 2016‐000095‐17), are currently recruiting. A similar study in which RDEB patients received transgenic autologous epidermal equivalents was recently performed at Stanford University.^85^

**Table 1:** Facts from JEB gene replacement therapies

Genetically corrected epidermal grafts *LAMB3* Showed regeneration of morphologically normal, fully differentiated and functional, mechanically stable and non‐blistering transgenic skin Can replace up to 80% of a patient's diseased skin^84^ Showed sustained synthesis of the transgenic protein^131^ Harbour EpSCs which expanded and ensured long‐term or permanent regeneration^84^ Despite the risk of insertional mutagenesis tumorigenicity has not been observed within a follow‐up period of currently more than 12 y (83, 84, 131, and unpublished observations) Following regeneration, there was no evidence of an immune response against the newly expressed protein (unpublished observations)

**Table 2:** Current strategies of molecular therapy in EB

Therapy		Principle	Current and past clinical trials	Study location, time, results
Gene therapy	Gene replacement (cDNA delivery)	Ex vivo gene transfer (viral or non‐viral mediated): skin cells are cultured, transduced with a vector encoding the functional cDNA copy of an EB‐causing gene and transplanted back *via* grafting of epithelial sheets or skin equivalents	JEB: Phase I—ex vivo grafting of gene‐corrected epidermal sheets with a retroviral vector expressing *LAMB3* cDNA, in 2 patients^82^, ^83^	2006, 2016 Austria/Italy Firmly adherent epidermis that proved stable for the duration of the follow‐up and beyond without any blisters, infections, inflammation or immune response
			JEB: Phase I/II—ex vivo grafting of gene‐corrected epidermal sheets with a gamma‐retroviral vector carrying *COL17A1* cDNA in 12 patients (HOLOGENE17)	Ongoing (initiated 04/2018) Austria
			RDEB: Phase I/IIa—ex vivo grafting of gene‐corrected LZRSE‐*COL7A1* engineered autologous epidermal sheets in 4 patients^85^	2010 USA (Stanford) Healing in some C7 gene‐corrected grafts, but the response was variable among patients and among grafted sites and generally declined over 1 y
			RDEB: Phase I/II—ex vivo grafting of gene‐corrected epidermal sheets with a gamma‐retroviral vector carrying *COL7A1* cDNA in 12 patients	Ongoing (initiated 01/2017) Austria
			Preclinical studies^87^, ^92^, ^95^, ^101^, ^132^	UK, France, Italy, USA
	Genome editing	Designer nucleases (eg CRISPR/Cas9, TALEN) directly target the mutated gene^133^, ^134^, ^135^	Preclinical studies^86^, ^88^, ^89^, ^90^, ^91^, ^93^, ^94^, ^96^, ^97^, ^98^, ^99^, ^103^, ^104^, ^110^	Austria, China, France, Italy, Spain, UK, USA
	Gene silencing	siRNA: knockdown of the mutant allele without silencing the wild‐type allele	Preclinical studies^136^	France
“Natural gene therapy”	Revertant mosaicism	Cultivation/graftingof cells in which the inherited mutation is corrected by a spontaneous genetic event (revertant cells)	JEB: Phase I—in vivo grafting of revertant epidermal sheets in two patients (*COL17A1* mutation, *LAMB3*)^137^, ^138^	2009, 2013 The Netherlands (Groningen) Few cells remained corrected in the graft; no clinically useful outcomes
Cell‐based therapy	Bone marrow (BM) stem cells	Transplantation (Tx) of allogeneic bone marrow (BMT): anti‐inflammatory effect; (supposed) theory that BM‐pluripotent stem cells can reprogramme to keratinocytes and home to the skin	RDEB: phase I—allogeneic hematopoietic cell Tx after an immune‐myeloablative chemotherapy in 7 patients (children)^139^	2007 USA (Minnesota) 5 patients showed improved wound healing and reduced blistering (30‐130 d after Tx) 2 deaths due to complications
			Severe EB: phase II—Biochemical correction of severe EB by allogeneic stem cell Tx (bone marrow or umbilical cord blood) and “off‐the‐shelf” mesenchymal stem cells in 75 patients	Ongoing (initiated 2010) USA (Minnesota)
			Severe EB: Phase II—Biochemical correction of severe EB by allogeneic cell Tx and serial donor mesenchymal stem cell infusions in 60 patients	Ongoing (initiated 2016) USA (Minnesota)
	Fibroblasts	Injection of WT allogeneic fibroblasts, to substitute the missing protein (C7 is synthetized by keratinocytes and fibroblasts)	RDEB: Phase I—single intradermal injections of allogeneic normal fibroblasts to 5 patients^140^	2008, UK Increased type VII collagen at the DEJ at 2 wk and at 3 mo following injection and increased anchoring fibrils (with altered morphology)
			RDEB: Phase II—intradermal injections of allogeneic fibroblasts in five patients, randomized controlled trial (RCT)^141^	2013, Australia All injected wounds (vehicle and allogeneic fibroblast) healed more rapidly compared to controls
			RDEB: Phase II—single intradermal injection of allogeneic fibroblasts into the margins of 26 chronic erosions in 11 patients (RCT)^142^	2013, UK All injected wounds healed initially (28 d) more rapidly than non‐injected wounds, without any difference between the cultured allogenic fibroblasts and vehicle
		Injections of autologous, gene‐corrected fibroblasts	RDEB: Phase I—intradermal injections of lentiviral‐mediated *COL7A1* gene‐modified autologous fibroblasts in 5 patients	Ongoing (initiated 2015) UK
			RDEB: Phase I/II—intradermal injections of lentiviral‐mediated *COL7A1* gene‐modified autologous fibroblasts (FCX‐007) in 6 patients	Ongoing (initiated 2016) USA (Stanford)
	Mesenchymal stem cells (MSC)	MSCs migrate to injured tissue and stimulate tissue regeneration; MSCs have anti‐inflammatory and immunomodulatory properties;	RDEB: Phase I—intradermal injections of allogeneic mesenchymal stromal cells in 2 patients^143^	2010, Chile At week 12, increased healing tendency compared to control sites. Clinical improvement lasted 4‐6 mo
			RDEB: Phase I—intravenous infusions of bone marrow‐derived non‐hematopoietic MSCs in 14 patients^144^	2015, Egypt Clinical improvement peaked in most of the patients after 3 mo; 2 patients showed improvement for at least 1 y
			RDEB: Phase I/II—intravenous allogeneic mesenchymal stromal cells in 10 children (EBSTEM)^145^	2013, UK Indications of reduced skin inflammation and improved wound healing; no increase in type VII collagen or new anchoring fibrils detectable in skin biopsies
			RDEB: Phase I/II—intravenous infusions of third party bone MSCs intravenous in 10 adults (ADSTEM)	Ongoing (initiated 2015) UK
			RDEB: Phase I/IIa—intravenous infusions of allogeneic ABCB5 + mesenchymal stem cells	Ongoing (initiated 10/2018) US (Minnesota), Germany
			DEB: Phase I/II—hydrogel sheet containing allogenic adipose‐derived mesenchymal stem cells (ALLO‐ASC‐DFU) in 5 patients	Ongoing (initiated 10/2015) Korea
	Induced pluripotent stem cells (iPSCs)	Reprogramming of somatic cells or revertant skin cells into iPSCs that can be grown and differentiated into any cell type (eg keratinocytes)	Preclinical studies^91^, ^92^, ^93^, ^104^, ^145^, ^146^, ^147^, ^148^	USA (Minnesota) Japan Austria Germany
RNA‐targeting therapy	Antisense oligonucleotide (AON)‐mediated exon skipping	Specific AONs bind to a mutant exon during the pre‐mRNA splicing process, whereby the exon is skipped resulting in a shortened transcript/protein	RDEB: Phase I/II—topical administration of QR‐313 (21‐nucleotide AON) in 8 patients with RDEB due to mutation(s) in exon 73 of *COL7A1* (RCT)	Ongoing (initiated 07/2018) US (Colorado)
	Spliceosome‐mediated RNA *trans*‐splicing (SMaRT)	Mutated region is replaced within a targeted pre‐mRNA by its wild‐type version by using the cell's own splicing machinery	Preclinical studies^79^, ^109^, ^113^, ^115^, ^116^, ^117^, ^118^, ^124^, ^149^, ^150^, ^151^	Austria
	Induced premature termination codon (PTC) read‐through	30% of *COL7A1* mutations are nonsense mutations, leading to PTCs and truncated C7 with diminished function. Small molecules (eg aminoglycosides, amlexanox) can induce PTC read‐through	JEB: Phase I/II—topical and intravenous gentamicin treatment in 6 patients with laminin 332 mutations	Ongoing (initiated 05/2018) US (California)
			RDEB: Phase I—topical and intradermal gentamicin treatment in 5 patients with *COL7A1* nonsense mutations^24^	2016, US (California) topical and intradermal gentamicin induced C7 and anchoring fibrils at the DEJ persisting for 3 mo; Topical gentamicin corrected DEJ separation, improved wound closure and reduced blister formation
			RDEB: Phase I/II—intravenous gentamicin treatment in 9 patients with *COL7A1* nonsense mutations	Ongoing (initiated 07/2018) US (California)
Protein therapy	Replacement/delivery of the aberrant or lacking protein	Topical application of transgenic HSV‐1	DEB: Phase I/II—Topical KB103 gel of non‐integrating, replication‐incompetent HSV‐1 expressing the human type VII collagen	Ongoing (initiated 05/2018) US (Stanford)
		Injection of C7	RDEB: Phase I/II—intravenous injection of collagen VII protein	Recruiting (initiated 11/2018) US (Stanford)
Small molecules	Diacerein	Inhibits the in vitro and in vivo production and activity of interleukin‐1β (IL‐1β) and other pro‐inflammatory cytokines	EBS‐gen sev: Phase I—topical diacerein 1% for the treatment of blisters in 5 patients	2013, Austria number of blisters was reduced significantly within 2 wk and remained below the initial level during withdrawal in 4 patients
			EBS: Phase II—long‐term safety and tolerability of diacerein 1% ointment	Ongoing (initiated in 2017) multi‐centre
			EB: Phase I—evaluation of the pharmacokinetics of diacerein and rhein and the safety of diacerein 1% ointment topically after maximum use	Ongoing (initiated 10/2018) US, France, Netherlands, UK
	AC‐203	Inhibits the production and activity of IL‐1β	EBS: Phase I—double‐blind, intra‐individual comparison, proof‐of‐concept trial of topical AC‐203 in 8 patients	Ongoing (initiated 04/2018) Taiwan
	Losartan, Ruxolitinib	Antifibrotic drug	Preclinical studies^152^	Germany
	Rigosertib	Polo‐like kinase 1 (PLK1) inhibitor	RDEB: Phase II—”First in EB” oral treatment for EB SCCS	Ongoing (initiated 08/2017) Austria

While replacement gene therapy is incapable to overcome dominant‐negative interference in the majority of EBS or DDEB forms, sustained expression of the functional transgene can replace the function of the endogenous non‐functional genes sufficiently to revert the phenotype in autosomal recessive diseases. An enduring replacement gene therapy relies on viral transfection of human epidermal stem cells with (full‐length) wild‐type cDNA. The protocol involves isolation of primary patient keratinocytes, which are expanded ex vivo in cell culture from a small skin biopsy. The presence of a sufficiently high number of epidermal stem cells (EpSCs) in the primary cultures prior to gene transfer and growth of transgenic cells into epidermal equivalents used for grafting accounts for permanent regeneration of a healthy, functional and renewing epidermis and extra‐/cellular environment. This can be a major challenge as EB patients may harbour a very limited number of EpSCs, which further decreases with the age of the patient as a result of continuous wounding and scarring.

As part of clinical studies involving gene therapy, several parameters need testing such as the efficiency of the viral gene transduction and safety with respect to genotoxicity. Finally, the transgenic cells, including EpSCs, are expanded and grown into cohesive epidermal sheets, which are grafted back onto a surgically prepared wound bed following established procedures similar to those used in the treatment of burn injuries.

While the vast majority of transduced keratinocytes are transit‐amplifying progenitors and differentiated keratinocytes, which are lost within weeks to few months after grafting, survival of a small number of long‐lived, extensively renewing transgenic EpSCs that produce progenitors replenishing terminally differentiated keratinocytes is sufficient to restore and sustain normal skin function and regeneration, when being transplanted onto wounded sites.^84^

Although this is not a systemic therapy, wound closure especially at sites of chronic and/or large blistering erosions can improve quality of life by reduction of itch, pain, inflammation, body fluid and protein loss, strengthening of microbial defense and prevention of cancer formation out of predisposing long‐standing wounds in junctional and dystrophic variants of EB. Therefore, ex vivo gene therapies based on highly efficient retroviral transduction of full‐length cDNA (*LAMB3*,* COL7A1*,* COL17A1*) to replace the function of the mutant endogenous genes have become a realistic treatment for generalized intermediate junctional and recessive dystrophic EB.

## 3 FROM REPLACEMENT GENE THERAPY TO PRECISION MEDICINE

Considerations to advance the above‐described strategy of cell and gene therapy include the employment of designer nucleases such as clustered regularly interspaced short palindromic repeats (CRISPR)/CRISPR‐associated protein 9 (Cas9) capable of mediating gene editing to correct distinct mutations at their endogenous loci. Instead of replacing the function of the mutant gene by adding a wild‐type cDNA, this technology allows the potentially traceless repair of the genetic mutation itself, eliminating the risk of insertional mutagenesis resulting from random transgene integrations.

### 3.1 Gene editing

Whereas retroviral transduction of a cDNA is highly efficient, the random integration of the transgene remains a risk. This risk can be eliminated by using the next‐generation gene therapy technology relying on designer nucleases. The nucleases are engineered to target the locus of interest specifically. They are transiently transfected and capable of inducing deletion or repair of disease‐causing mutations.

Most efforts to develop gene editing–based therapies for EB have focused on the correction of RDEB or DDEB mutations in *COL7A1,* 86, 87, 88, 89, 90, 91, 92, 93, 94, whereas JEB (*LAMA3* 95 and *LAMB3* 96) and EBS (*KRT5* 97, *KRT14* 98) were less frequently reported. In contrast to replacement gene therapies, where one vector can be used to treat all EB patients with recessive mutations in a specific gene, most designer nuclease‐mediated approaches that aim to repair gene defects are patient‐ or rather mutation‐specific. The endogenous DNA damage repair mechanisms triggered and harnessed by the technology require that designer nucleases target specific DNA sequences in close proximity to the mutation site. For genes such as *COL7A1* with a size of >30 kb and disease‐causing mutations spread almost along the entire length of the gene, this is challenging. Among the first targets chosen from our laboratory and others to develop efficient nucleases were therefore hot‐spot mutations such as the c.6527insC in exon 80 of *COL7A1*, which is highly prevalent in the Spanish dystrophic EB patient population.^86^, ^88^, ^94^, ^99^

Dominant‐negative mutations such as those common in EBS can be corrected in different ways. In addition to a mutation‐specific repair approach for a *KRT14* mutation,^98^ we developed another treatment approach based on deletion of a mutant keratin 5 allele while leaving the wild‐type allele intact and taking over the function.^97^ The latter study demonstrated that one gene‐specific designer nuclease could be employed to inactivate any EBS‐causing mutant *KRT5* allele by inducing insertions or deletions (indels) via the error‐prone non‐homologous end‐joining (NHEJ) repair pathway causing a frameshift at a site within the target gene that is independent on the location of the disease‐causing mutation.

First attempts of gene editing mutations associated with EB used meganucleases.^89^ These were soon replaced by approaches employing the more specific transcription activator‐like effector nucleases (TALEN).^86^, ^91^, ^97^, ^99^ Due to the straightforward use and rapid advancement of the technology, CRISPR is today dominating the gene editing field. Whereas most CRISPR approaches harnessed the DNA double‐strand break inducing *Streptococcus pyogenes* Cas9 (SpCas9) nuclease variants, we have recently developed double nicking approaches based on single‐strand DNA breaks (nicks) at the targeting site with the intention to reduce off‐target site effects.^88^, ^98^ Compared to the extremely high numbers of off‐target site modifications inherent to replacement gene therapies resulting from multiple random integrations in the treated cells, off‐target mutations are rare or non‐existent upon designer nuclease treatment.^100^

While most gene editing approaches published to date for EB used viral transfer of the nuclease or the repair template or both,^86^, ^87^, ^89^, ^92^, ^94^, ^95^, ^96^, ^101^, ^102^, ^103^, ^104^, ^105^ we are focusing on the development of non‐viral delivery^88^, ^98^ and traceless targeting^97^ approaches with a view to translation into the clinic. Although non‐viral keratinocyte transfection is less efficient and, instead of nearly 100% viral transduction efficiency, reaches maximally 70% efficiency, it avoids the risk of insertional mutagenesis. Novel safer non‐viral transfer methods are currently being developed.^106^, ^107^, ^108^ Like replacement gene therapy, the majority of the current gene editing approaches aim for ex vivo therapy often including a selection step before grafting of corrected cells. We showed that plasmid DNA can be delivered and expressed following gene gun delivery into the skin of mice.^109^ Wu et al^110^ demonstrated data where they successfully targeted EpSC in vivo by electroporation of RNPs, consisting of CRISPR/Cas9 and gRNA, in an RDEB mouse model.

Gene therapy targeting keratinocytes and/or fibroblasts^87^, ^101^ allows treatment of patches or larger areas of the skin. The severe types of EB, however, also involve internal organs, demanding a systemic approach.

### 3.2 RNA editing

Spliceosome‐mediated *trans*‐splicing (SMaRT) technology allows correction of dominant or recessive mutations at mRNA level. Specifically designed RNA *trans*‐splicing molecules (RTMs) bind to the target pre‐mRNA and replace mutant exons by wild‐type sequences. RTMs are capable of replacing large parts of a transcript and a single type of therapeutic molecule could therefore be used to treat patients with several mutations.^109^, ^111^ Because SMaRT targets RNA rather than DNA, the corrections are not permanent and the risk of mutagenesis is low. The transient nature of the correction at mRNA level however means that the treatment has to be repeated to result in an improvement of the disease phenotype. In the case of collagen type VII with a predicted half‐life of several weeks,^112^ the effect of an application can be relatively long lasting. We demonstrated delivery of functional RTMs into murine epidermis and upper dermis with a gene gun.^109^ Specific RTMs for several genes involved in EB have been developed at the EB House Austria (*COL7A1*,* Col17A1*,* KRT14* and *PLEC* 79, 88, 113, 114, 115, 116, 117, 118) and holds US and European patents on improved RTMs and their uses (US8735366, EP2320952).

Some EB‐causing mutations reside in exons that are non‐essential for the function of the protein that is encoded by the gene.^119^ Within *COL7A1,* exons 79, 73 and 80 are dispensable and can, in the presence of specifically designed antisense oligonucleotides (ASOs), be skipped during pre‐mRNA splicing.^120^, ^121^, ^122^ Furthermore, ASOs can be employed to modulate and improve SMaRT efficiency.^123^, ^124^ With approximately 21 nucleotides in length, ASOs are relatively small and can be chemically modified to increase their stability. ASO therapeutics has recently been approved in the United States for Duchenne muscular dystrophy and in the United States and Europe for spinal muscular atrophy. An exon 73‐skipping ASO trial for RDEB is currently recruiting patients in the United States.

## 4 CONCLUSIONS AND FUTURE CHALLENGES OF EB RESEARCH

Epidermolysis bullosa research has advanced considerably in the past decade (Table 3), and wound and pain management has improved. Inclusion of several clinical disciplines in addition to dermatology has proven essential in order to provide comprehensive care for EB patients. Novel highly efficient cancer therapies give hope in the battle to fight the aggressive SCCs in the most severe EB forms. Guidelines on oral health care,^125^ pain management,^126^ wound care^127^, ^128^, ^129^ and cancer management^130^ were compiled and made available. Networks connecting clinicians, patients and caregivers have now formed worldwide. The EB2018 Research Conference hosted in Salzburg by the EB House and organized by its EB‐CLINET branch highlighted recent progress aimed at improving EB patient's quality of life.^4^ A variety of new research areas with relevance to EB were also represented. EB is clearly not restricted to the visibly affected skin but has to be regarded as a severe systemic disease requiring input from a large range of specializations. The impact of chronic inflammation, fibrosis and the microbiome on the development of SCCS in the most severe forms of EB has become apparent, and a growing number of promising new targets for treatments are emerging. Strong efforts are being made to set up a registry that allows identification of suitable candidates for essential clinical trials despite the fact that EB is a rare disease.

**Table 3:** Comparison of EB therapy approaches

	Gene therapy		Cell therapy			RNA‐targeting therapy			Protein therapy (g)
	Gene replacement (a)	Gene editing (b)	Revertant cell therapy (mosaicism) (c)	BM‐SC Tx (d)	Other cell types (fibroblasts, MSCs) (e)	AON	PTC	SMaRT	
Advantages									
Systemic application feasible	−	−/+	−	+	−/+	+	+	−	+
Long‐term correction	+	+	+	−	−	−	−	−	−
Specific (gene/sequence) targeting	−	+	−	−	−	+	−	+	−
Personalized therapy	−	+/−	+	−	−	+	+	+	+
Applicable for									
Recessive EB forms	+	+	+	+	+	+	+	+	+
Dominant EB forms	−	+	+/−	N/A	N/A	+	−	+	(+)
Combination therapy reasonable with	(e)	(e), iPSC^a^	iPSC	−	(a), (b), iPSC	−	−	−	−
Limitations									
Tumorigenesis	+ + (insertional mutagenesis)	−	−	+ (immunosuppression)	+/− (immunosuppression)	unk	unk	unk	−
Estimated risk of off‐target effects	++	+	−	−	−	+	+	+	−
Risk of adverse immunological response	+	+	−	+	+	+	+	+	+
Major side effects	−	unk	−	+ (procedure related)	−	unk	unk	unk	unk
Invasiveness	+	+	+	+	−	−	−	−	−
High degree of complexity	++	++	+	+	−	−	−	−	−
Efficiency	++	+	N/A	N/A	N/A	+	N/A	N/A	N/A

Abbreviations: ++, to a very great extend; +, largely true; +/−, partly true; −, not true, unk, unknown; N/A, not applicable, (+), theoretically possible.

a iPSC, induced pluripotent stem cells, differentiated into other cell types (eg keratinocytes, fibroblasts).

The molecular tools that allow micro‐surgery to correct disease‐causing mutations within the affected genes were discovered only recently and have since been continuously improved. Gene editing technology is breaking new grounds of research with the prospect of devising permanent, safe, tolerable and feasible delivery of local and possibly systemic treatments for inherited diseases. Applied at an early stage, efficient gene therapies may prevent the development of symptoms and at later stages prevent disease progression involving the development of devastating chronic EB lesions and life‐threatening cutaneous SCC. There are still major challenges but also exciting discoveries ahead.

## CONFLICT OF INTEREST

JWB owns shares of the company Diaderm, which receives licence payments from Castle Creek Pharma (CCP). CCP is conducting a phase 2/3 study with a diacerein‐containing ointment (NCT03154333). JWB was holder of the EMA orphan drug designation on diacerein in epidermolysis bullosa.

## AUTHOR CONTRIBUTIONS

CP, JR and ML wrote the original draft. CP, JR and ML and JWB reviewed and edited the manuscript. All authors have read and approved the final manuscript.
